# Interplay between circadian rhythms, gut microbiota, and MASLD: from mechanistic foundations to therapeutic opportunities

**DOI:** 10.3389/fmed.2026.1767462

**Published:** 2026-04-15

**Authors:** Yu-Jia Chen, Bo-Wen Yang, Zun-Cai Gu, Jun Han

**Affiliations:** 1 Department of Cardiology, Affiliated Huishan Hospital of Xinglin College, Nantong University, Wuxi, Jiangsu, China; 2 Department of Hepatology, Affiliated Wuxi Fifth Hospital of Jiangnan University, Wuxi, Jiangsu, China

**Keywords:** circadian rhythm, gut microbial dynamics, gut microbiota, liver axis, metabolic related fatty liver disease

## Abstract

Metabolic dysfunction-associated fatty liver disease (MASLD), previously known as non-alcoholic fatty liver disease (NAFLD), has become the most common chronic liver disease worldwide. Although excessive lipid accumulation, insulin resistance, and chronic low-grade inflammation are recognized as the main pathophysiological drivers, an increasing body of research indicates that the relationship between circadian rhythms, gut microbiota, and liver metabolism is far more complex than previously imagined, forming a systemic regulatory network. Disruption of circadian rhythms can affect the temporal coordination of metabolic pathways in the liver and other surrounding tissues. At the same time, the gut microbiota itself also exhibits circadian rhythm variations. The dysregulation of these rhythms, leading to microbial imbalance, intestinal permeability defects, and imbalances in microbial metabolites, can exacerbate lipid deposition and inflammatory responses in the liver. Research shows that important microorganisms can produce short-chain fatty acids, regulate bile acid balance, and enhance intestinal barrier function, creating a synergistic effect with the host's circadian rhythms. Conversely, during circadian disruption, the proliferation of harmful symbionts can exacerbate the entry of lipopolysaccharides into the bloodstream, oxidative stress, and the development of steatohepatitis. This relationship among the three establishes the ' circadian rhythm-gut microbiota-liver axis' as a new model for understanding the mechanisms underlying MASLD and for developing temporal therapies and microbiome interventions. This review systematically explores how circadian rhythms regulate the relationship between the gut microbial ecology and liver metabolism, focusing on the microbial species closely related to the interaction between circadian rhythms and MASLD. It also introduces emerging therapeutic strategies, including time-restricted feeding, circadian probiotics, postbiotics supplementation, and circadian rhythm drugs. These findings collectively suggest that targeting the temporal dimension of the interactions between the host and microbiota holds clinical potential for the prevention and treatment of MASLD.

## 1 Introduction

Metabolic dysfunction–associated steatotic liver disease (MASLD) has rapidly become a global health concern, affecting nearly one-quarter of the adult population and posing a major risk for liver fibrosis, cirrhosis, hepatocellular carcinoma, and cardiovascular complications (1). Formerly known as non-alcoholic fatty liver disease (NAFLD), MASLD has been reclassified to highlight its strong association with metabolic dysfunction, rather than alcohol consumption alone, reflecting an evolving understanding of its multifactorial etiology (1, 2). NAFLD was once the predominant term describing excessive fat deposition in hepatocytes. However, due to its reliance on exclusionary diagnosis and lack of etiological specificity, the international academic community proposed replacing it with MASLD in 2023. Despite growing clinical interest, there remains a significant lack of approved therapies capable of effectively halting fibrosis progression in metabolic dysfunction-associated steatotic liver disease (MASLD) (3). While several antidiabetic agents—such as GLP-1 receptor agonists and SGLT2 inhibitors—have demonstrated the ability to reduce hepatic steatosis in patients with type 2 diabetes or obesity, evidence supporting their antifibrotic efficacy is still limited and its management remains limited to lifestyle modifications such as weight reduction and dietary interventions (4, 5). This gap underscores the urgency of identifying novel mechanistic drivers and therapeutic targets that account for MASLD’s systemic and dynamic nature.

Recent years have seen a paradigm shift in MASLD research, moving beyond static metabolic abnormalities toward a more integrated view that includes endocrine, immunological, and chronobiological inputs. One of the most compelling developments in this field is the recognition of circadian rhythms—the endogenous 24-h timing system—as a central coordinator of organismal physiology and metabolism (6–8). Circadian clocks operate not only in the brain’s suprachiasmatic nucleus (SCN), which responds to light cues to regulate sleep–wake cycles, but also in peripheral tissues such as the liver, adipose tissue, pancreas, and gastrointestinal tract (9–11). These tissue clocks orchestrate rhythmic expression of genes involved in glucose and lipid metabolism, oxidative stress responses, bile acid synthesis, and inflammatory signaling (11–13). Disruption of these rhythms, as seen in shift workers, individuals with sleep disorders, and populations exposed to irregular eating schedules, is strongly associated with obesity, insulin resistance, and fatty liver disease (8, 14, 15).

Parallel to circadian biology, the gut microbiota has emerged as a crucial determinant of metabolic health (16). Once thought to be a relatively stable community, the gut microbiota is now known to undergo diurnal oscillations in composition, function, and metabolite production (17, 18). These microbial rhythms are entrained by host feeding patterns, hormonal fluctuations, and gastrointestinal motility cycles. Importantly, microbial metabolites such as short-chain fatty acids (SCFAs), bile acid derivatives, and indoles exert profound effects on hepatic energy metabolism, inflammation, and circadian gene expression (18–20). Dysbiosis—broadly defined as an imbalance or loss of rhythm in microbial ecology—has been consistently linked to MASLD progression through multiple pathways, including increased intestinal permeability, endotoxin spillover, and impaired bile acid signaling.

As a result, a conceptual framework has emerged that positions MASLD as a disorder driven not only by nutrient overload and metabolic impairment, but also by the destabilization of a tripartite axis involving the circadian system, gut microbiota, and liver physiology. This interdependent system highlights the liver’s central role as both a metabolic buffer and a chronobiological hub, receiving signals from the central clock and gut-derived metabolites while exerting feedback on systemic metabolism through bile acids, glucose flux, and inflammatory mediators. Disruption at any of these nodes—whether circadian misalignment, dietary irregularity, or microbial dysbiosis—can propagate pathological changes across the network, amplifying liver injury and metabolic dysfunction.

This review integrates recent advances to elucidate the complex interplay between circadian rhythms, gut microbiota, and metabolic liver function in MASLD. It first examines the role of the circadian clock in orchestrating hepatic metabolic pathways, highlighting how temporal gene expression patterns govern processes such as lipid storage, glucose homeostasis, and bile acid cycling. The discussion then turns to the gut microbiota, exploring how its composition and metabolic output exhibit robust diurnal oscillations that are entrained by feeding patterns and, in turn, modulate host circadian and metabolic states. Special emphasis is placed on key microbial taxa that are responsive to circadian cues and contribute directly to MASLD progression or protection. Finally, the review outlines emerging therapeutic strategies that target the circadian–microbiome–liver axis, including time-restricted nutrition, chronobiotic probiotics, postbiotic metabolites, and circadian-timed pharmacotherapy. By weaving together these perspectives, the review aims to advance an integrated framework for understanding MASLD pathogenesis and to inspire new chronotherapy-based approaches in metabolic liver disease management (Table 1).

**Table 1:** Summary of key gut microbial taxa involved in circadian regulation and MASLD pathogenesis.

Genus	Circadian features	Key functions (brief)	Effects in MASLD (evidence summary)	Evidence Type (Animal/Clinical/Mechanism Studies)	MASLD net effect
*Akkermansia muciniphila*	Activity linked to feeding cycles; depleted by circadian misalignment and high-fat diet.	Mucin degradation/renewal; strengthens mucus barrier and tight junctions; stimulates GLP-1; modulates bile-acid signaling.	Reduced abundance in MASLD/NAFLD; supplementation (live or pasteurized, select strains) reduces hepatic fat and inflammation in animals and showed metabolic benefits in early human proof-of-concept work.	Animal Mechanisms and a Human Exploratory Supplement Trial (Safety and Evidence of Metabolic Improvement)	Protective
*Faecalibacterium prausnitzii*	Butyrate production peaks after feeding; oscillations attenuated by HFD and circadian disruption.	Major butyrate producer — fuels colonocytes, activates AMPK, and acts via HDAC inhibition to modulate transcription and inflammation.	Depleted in NAFLD/MASLD cohorts; butyrate or *F. prausnitzii* supplementation improves hepatic steatosis and inflammatory markers in preclinical models.	Mouse models and several in vitro/preclinical studies, as well as studies involving administration of a limited number of strains	Protective
*Lactobacillus*	Abundance and SCFA production typically peak during the host’s active/feeding phase; disrupted by jet-lag, constant-light or HFD.	Produces lactate/acetate (cross-feeding to butyrate producers); many strains express BSH; enhances epithelial tight junctions and incretin responses.	Strain-specific hepatoprotective effects (e.g., *L. rhamnosus* GG reduces fructose-induced steatosis in mice); clinical findings heterogeneous.	Multiple animal interventions and several clinical trials/reviews	Protective
*Bifidobacterium*	Feeding-entrained oscillation; sensitive to host timing signals (e.g., melatonin/feeding schedule).	Produces acetate/propionate/lactate and indole derivatives; activates GPR41/43, AhR and TLR2; supports barrier integrity.	Often reduced in MASLD cohorts; specific strains or synbiotic combinations (*B. breve, B. longum*) reduce hepatic fat and ALT/AST in animal models and some clinical trials.	Multiple animal models (HFD) and some clinical/review evidence	Protective
*Bacteroides*	Postprandial abundance peak; tightly coupled with bile-acid flux and feeding cycles.	Polysaccharide degradation; widespread BSH activity; produces propionate/acetate; modulates FXR/TGR5 signaling.	Context dependent: supports host metabolism under homeostasis but in dysbiosis can increase hydrophobic secondary bile acids (e.g., DCA), suppress FXR-FGF19 feedback and promote hepatic lipogenesis and inflammation.	Animal and Human Association Studies: A Review of Mechanisms in Bile Acid Metabolism	Situational-dep
*Enterobacteriaceae*	Likely feeding-linked (resistant-starch niche); time-series data limited.	Keystone resistant-starch degrader (*R. bromii*); promotes cross-feeding to butyrogenic taxa and supports SCFA pools.	Some strains (e.g., *R. faecis*) show antifibrotic/hepatoprotective effects in models; genus heterogeneity (e.g., *R. gnavus* can be pro-inflammatory).	Population microbiome association studies and animal causality experiments involving transplantation/strain administration (HiAlc *K. pneumoniae*-induced hepatic steatosis).	Situational-dep
*Ruminococcus*	Expand under circadian disruption, barrier impairment, or HFD.	LPS production (TLR4 → MyD88 → NF-κB activation); some strains produce endogenous ethanol (HiAlc *Klebsiella*).	Enriched in MASLD; associated with elevated portal/systemic LPS, hepatic inflammation, oxidative stress via CYP2E1 and fibrosis; HiAlc *Klebsiella* can induce NAFLD in mice.	Basic ecological research, animal supplementation studies, and human population associations (fiber processing/fiber supply related)	Pathogenic
*Desulfovibrio*	Enriched by HFD and circadian disruption; responsive to sulfur substrate availability.	Sulfate reduction → H₂S production; excessive H₂S can erode mucus and disrupt tight junctions.	Associated with increased gut permeability, oxidative stress and worsened liver injury in animal and some human studies; suppression improves barrier and hepatic outcomes in preclinical work.	Multiple animal models and recent reviews and mechanism studies (H₂S and liver inflammation/autophagy association)	Pathogenic
*Alistipes*	Diurnal abundance data limited; microbiota-derived tryptophan metabolites (e.g., IPA) show relatively stable serum patterns that may interact with host rhythms.	Produces indole derivatives (IPA/IAA) that activate AhR/PXR; may support barrier and anti-inflammatory signalling; some strains participate in butyrate cross-feeding.	Reduced in MASLD; *A. putredinis* supplementation (Ap77) reduced hepatic fat and inflammation in rodent models; cohort data link IPA to fibrosis phenotypes (context-dependent effects reported).	Recent animal supplementation studies and clinical metabolite association research (IPA negatively correlated with liver fibrosis)	Situational-dep

## 2 Circadian system and hepatic metabolic regulation

Circadian rhythms are intrinsic, roughly 24-h oscillations that enable organisms to anticipate and adapt to recurring environmental changes, such as light–dark cycles and nutrient availability (21). In mammals, this temporal coordination is achieved through a hierarchical circadian timing system composed of a master clock located in the suprachiasmatic nucleus (SCN) of the hypothalamus and multiple peripheral clocks distributed across metabolic organs, including the liver (22, 23). The SCN synchronizes peripheral oscillators through behavioral cues, neuroendocrine signals, and body temperature rhythms, while each peripheral clock independently regulates local physiological processes in a time-of-day–dependent manner. Among these organs, the liver stands out for its high degree of rhythmic transcriptional and metabolic activity, positioning it as a key mediator of whole-body metabolic homeostasis (24).

### 2.1 Molecular architecture of the circadian clock in the liver

At the cellular level, circadian rhythm generation relies on a set of transcription–translation feedback loops (TTFLs) that form the molecular clockwork. Central to this system is the CLOCK: BMAL1 heterodimer, which binds to E-box sequences in the promoters of clock-controlled genes, activating their rhythmic expression (25). Key among these targets are the Period (PER1, PER2, PER3) and Cryptochrome (CRY1, CRY2) genes, whose protein products accumulate over the day, eventually forming complexes that inhibit CLOCK: BMAL1 transcriptional activity, thereby closing the feedback loop (26). A secondary stabilizing loop involves REV-ERB and ROR nuclear receptors, which regulate BMAL1 transcription and thus ensure robustness and precision of the circadian cycle (27, 28).

**Figure 1:**
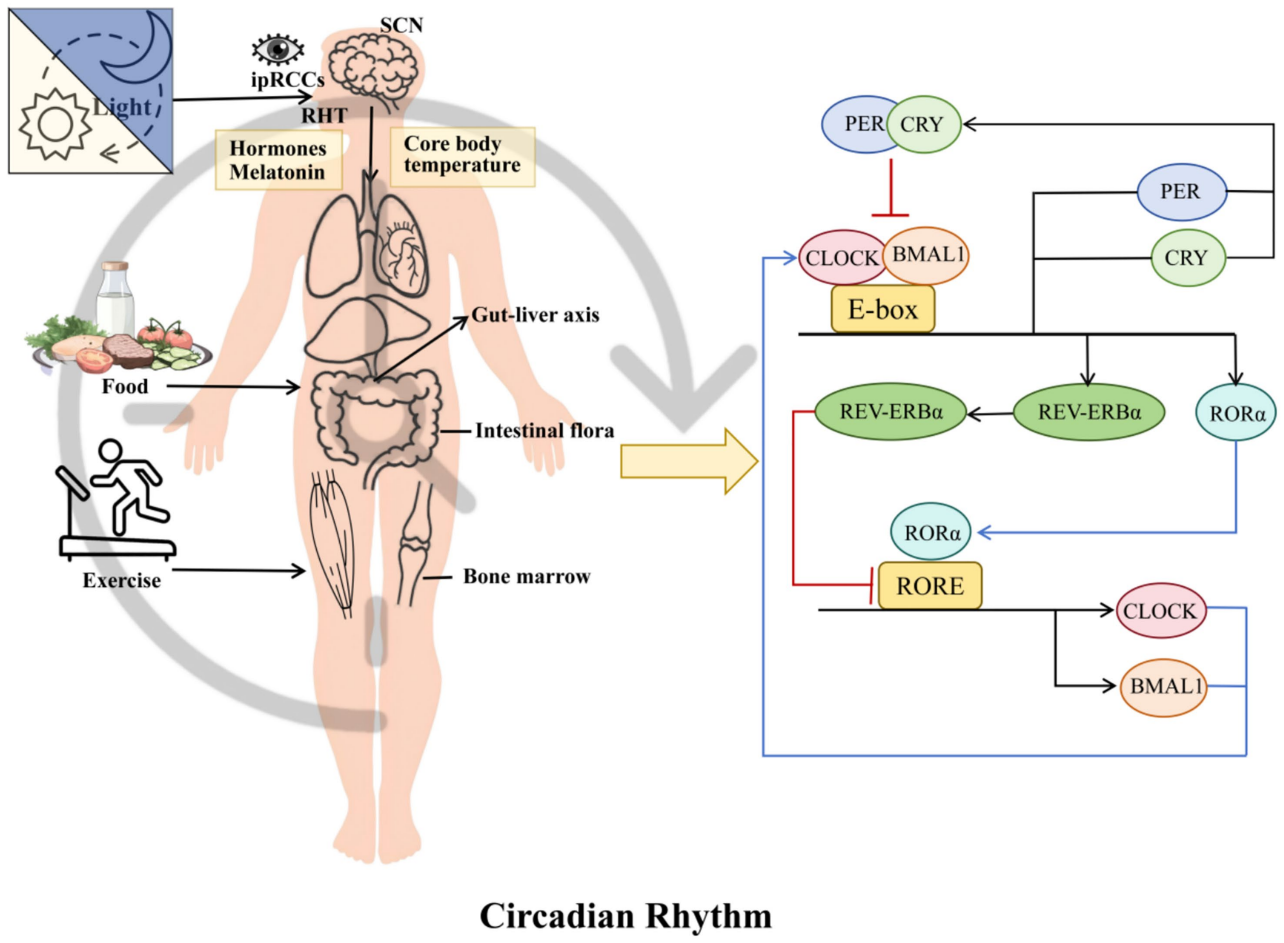
Overview of the circadian rhythm system and its multiorgan regulatory network.

**Figure 2:**
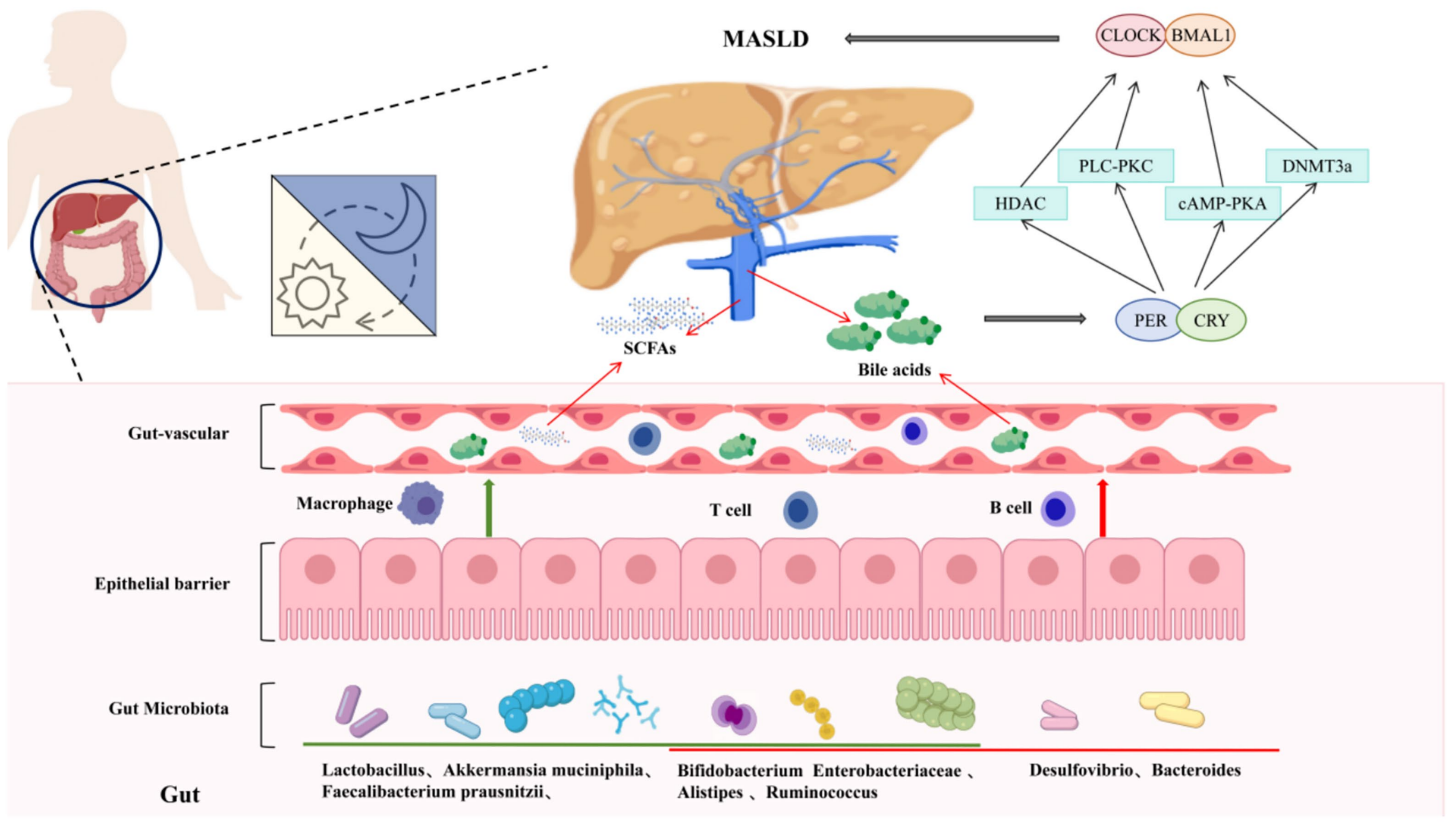
Interactions between circadian rhythm, gut microbiota, and the gut–liver axis in MASLD pathogenesis.

In hepatocytes, this molecular clock governs a large proportion of the transcriptome—estimates suggest that over 50% of liver genes exhibit circadian rhythmicity under physiological conditions (29). Notably, many of these genes orchestrate lipid metabolism, glucose handling, detoxification, bile acid synthesis, and redox balance. For instance, lipogenic genes such as SREBP-1c and FASN peak during the active feeding period, promoting lipid storage when nutrients are abundant, whereas fatty acid oxidation genes such as CPT1a reach peak expression during the fasting phase, supporting energy mobilization. Similarly, gluconeogenic enzymes (PEPCK, G6Pase) and bile acid synthetic enzymes (CYP7A1) display rhythmic expression patterns that ensure metabolic processes occur at the most suitable time of day (30, 31) (Figures 1, 2).

This temporal partitioning of metabolic tasks is essential to prevent cellular stress, balance substrate utilization, and maintain hepatic metabolic health. Disruption of clock function—either through genetic manipulation or behavioral circadian misalignment—rapidly leads to metabolic dysregulation and predisposes to liver disease.

### 2.2 Circadian disruption as a driver of metabolic liver disease

Growing evidence suggests that circadian disruption is a critical risk factor for the development and progression of MASLD (7, 32). Environmental factors such as shift work, irregular eating schedules, artificial light exposure at night, and chronic sleep deprivation can desynchronize central and peripheral clocks, leading to metabolic imbalance (6, 8, 14, 15, 33). BMAL1-deficient mice have several metabolic defects, including high serum triglycerides (TAG) (34–36) and low circulating insulin associated with high insulin sensitivity (34, 37, 38). Associated with these metabolic defects, they present signs of early aging that start around 18 week (39).

In addition to BMAL1, multiple other core clock components have been mechanistically linked to MASLD. Mice carrying the dominant-negative *Clock* mutation develop obesity, hypertriglyceridemia, and marked hepatic steatosis driven by persistent activation of SREBP-1c–mediated lipogenesis (40). Studies using Per1/Per2 double-knockout mice report blunted circadian oscillations of hepatic lipid-related transcripts and altered mitochondrial oxidative rhythms, with transcriptomic and functional evidence that PER proteins temporally gate hepatic lipid handling and fat absorption (41). Similarly, Cry1/Cry2 double-knockout animals exhibit diet-sensitive metabolic instability—including hyperinsulinemia, glucose intolerance, and increased hepatic lipid accumulation under high-fat feeding—consistent with a role for CRY proteins in repressing anabolic signaling and maintaining hepatic metabolic rhythm (42).

Nuclear receptors that function as auxiliary clock regulators further contribute to MASLD pathogenesis. Deletion of *Rev-erbα (Nr1d1)* leads to derepression of lipogenic and inflammatory gene networks, resulting in uncontrolled hepatic lipid accumulation and a more rapid transition toward steatohepatitis (43). Conversely, loss of *RORα* disrupts bile acid rhythmicity, impairs FXR–FGF15 signaling, reduces fatty acid oxidation, and produces severe hepatic steatosis, particularly under metabolic stress (44). Upstream kinases that determine clock periodicity also influence liver fat accumulation: disruption of casein kinase 1δ/*ε*—key regulators of PER protein stability—abolishes rhythmic lipid metabolic oscillations and increases vulnerability to high-fat-diet–induced steatosis (45, 46).

Additional studies have shown that transcription factors downstream of the clock also modulate hepatic lipid metabolism. Casein-kinase–dependent destabilization of clock repressors, altered DBP-driven transcriptional rhythms, and impaired PPARα–clock crosstalk each compromise *β*-oxidation and bile acid turnover, further predisposing animals to MASLD (47).

Collectively, these findings indicate that disruption of core or auxiliary circadian regulators—whether BMAL1, CLOCK, PER, CRY, REV-ERBα, RORα, or CK1 isoforms—leads to a convergent phenotype characterized by impaired temporal organization of hepatic metabolism. The resulting loss of coordinated oscillations in lipid synthesis, *β*-oxidation, bile acid turnover, oxidative stress management, and insulin signaling creates a metabolic environment highly permissive to hepatic fat accumulation, inflammation, and injury. These observations support the concept that MASLD is fundamentally a disease of circadian misalignment, in which the timing of metabolic processes is as crucial as their magnitude.

## 3 Circadian rhythm and gut microbial dynamics

The gut microbiota is increasingly recognized as an ecosystem governed not only by taxonomic composition and functional capabilities but also by oscillatory behavior that reflects and interacts with host circadian rhythms (48, 49). Under physiological conditions, the gut microbiome undergoes diurnal fluctuations in composition, abundance, metabolic output, and gene expression, shaped primarily by feeding patterns and associated host signals. This section explores how microbial rhythms arise, how they are perturbed by circadian misalignment, and how they participate in the gut–liver metabolic axis relevant to MASLD (50–52).

### 3.1 Diurnal oscillation patterns in gut microbiota

The intestinal flora is mainly divided into symbiotic bacteria (intestinal dominant flora), opportunistic pathogens (pathogenic symbionts), and transient bacteria. A growing body of research has demonstrated that the gut microbiota exhibits rhythmicity at several organizational levels (48, 49, 51, 52). In healthy animals and humans, a subset of bacterial taxa—including beneficial fermentative genera such as *Lactobacillus*, *Faecalibacterium*, and *Bifidobacterium*—tend to peak during periods of active feeding, reflecting substrate availability and synchronized metabolic demand (53, 54). Conversely, taxa such as *Enterobacteriaceae* may gain a transient relative advantage during fasting, as luminal oxygen levels shift in their favor.

These compositional changes are coordinated with rhythmic fluctuations in microbial metabolic activity. Short-chain fatty acids (SCFAs), particularly acetate and butyrate, show peak production in response to feeding and act as signaling molecules that modulate hepatic lipid oxidation, glucose metabolism, and even the transcriptional activity of clock-related genes (55). Similarly, the microbial conversion of primary bile acids to secondary bile acids exhibits a clear temporal pattern, indicating an interdependent relationship between dietary cues, host circadian biology, and microbial functionality (55).

### 3.2 Impact of circadian disruption on microbial composition and function

Circadian disruption—caused by irregular sleep, shift work, jet lag, or mistimed food intake—can rapidly perturb the structure and rhythmicity of the gut microbiota. In rodent studies, even a single episode of circadian misalignment results in diminished microbial oscillations, decreased abundance of SCFA-producing taxa, and shifts toward pro-inflammatory genera such as Enterobacteriaceae and *Desulfovibrio*. These changes are accompanied by reductions in butyrate levels, alterations in bile acid pools, and impaired epithelial barrier integrity (55).

In clinical settings, similar consequences are observed in night-shift workers and individuals exposed to recurrent circadian misalignment. These populations exhibit reduced microbial diversity, elevated markers of metabolic endotoxemia, and increased circulating inflammatory cytokines—all of which correlate with greater incidence of insulin resistance, hepatic lipid accumulation, and progression toward MASLD (8, 14, 15, 56).

Importantly, such dysbiosis is not just a secondary outcome of behavioral disruption: it feeds forward into metabolic and inflammatory pathways, exaggerating hepatic stress and promoting disease progression.

### 3.3 Gut microbiota as a mediator in the circadian–liver Axis

The gut microbiota plays a pivotal integrative role within the circadian–liver axis, translating rhythmic dietary and behavioral cues into metabolic signals that influence hepatic function (49). Oscillations in microbial composition and activity generate time-of-day–dependent patterns in key metabolites, many of which reach the liver through the portal circulation. Short-chain fatty acids, particularly acetate and butyrate, rise in concert with feeding cycles and exert broad regulatory actions that support metabolic homeostasis, including the promotion of fatty acid oxidation and the attenuation of lipogenic pathways (57). Similarly, the microbial conversion of bile acids into their secondary forms follows a diurnal pattern and provides a mechanism through which gut bacteria modulate FXR- and TGR5-dependent signaling in the liver (52, 58). Through these metabolite-driven interactions, the microbiota helps coordinate hepatic lipid metabolism, glucose handling, and inflammatory responses with the temporal structure of daily living. As such, the gut microbiome represents a critical intermediary that reinforces circadian alignment across organ systems, while its disruption may contribute to the metabolic vulnerabilities characteristic of MASLD (59). Furthermore, microbial-derived lipopolysaccharides (LPS) released during dysbiosis can breach a compromised gut barrier and trigger Toll-like receptor 4 (TLR4) signaling in Kupffer cells, leading to cytokine release, hepatic lipid deposition, and fibrosis. Such interactions underscore the microbiome’s unique role as a mediator in both physiological and pathological states of the circadian–liver axis (60).

Emerging evidence indicates that restoring circadian homeostasis through time-restricted feeding or sleep normalization can partially rescue microbial rhythmicity and improve hepatic outcomes. This reinforces the concept that microbial rhythms are both responsive to and regulatory of circadian-driven metabolic processes (53, 61).

### 3.4 Direct regulatory mechanisms of gut microbiota-derived metabolites on Core clock genes in hepatocytes

The autonomous oscillatory loop composed of core clock genes in hepatocytes serves as the primary regulator of hepatic metabolic rhythms. Recent studies have demonstrated that metabolites derived from gut microbiota, particularly SCFAs, along with endogenous hepatic metabolites, can precisely modulate the transcription and expression of core clock genes. This modulation occurs through direct binding to receptors, regulation of epigenetic modifications, or activation of downstream signaling pathways.

Short-chain fatty acids—primarily acetic acid, propionic acid, and butyric acid—are key products of gut microbiota fermentation of dietary fiber. These acids can directly reach the liver via the portal vein and influence the expression of circadian clock genes in hepatocytes through both receptor-dependent and non-receptor-dependent pathways. Butyric acid and propionic acid demonstrate high-affinity binding to G protein-coupled receptors (GPR41 and GPR43) located on the surface of hepatocytes. This binding activates the downstream phospholipase C-protein kinase C (PLC-PKC) signaling pathway, promoting the phosphorylation of the transcription factor cAMP response element-binding protein (CREB). The phosphorylated CREB binds directly to the cyclic adenosine monophosphate (cAMP)-responsive element (CRE) in the promoter regions of the BMAL1 and PER2 genes, significantly enhancing their transcriptional activity (62–64).

*In vitro* experiments have shown that treatment with 10 mM butyric acid in human hepatocyte cell lines (HepG2) increases BMAL1 mRNA expression by 2.3-fold and PER2 expression by 1.8-fold; these effects can be inhibited by a specific GPR43 antagonist. Additionally, as a histone deacetylase (HDAC) inhibitor, butyric acid enters the nucleus of hepatocytes and inhibits the deacetylation of histone H3K9 by HDAC1/3, leading to chromatin relaxation in the promoter regions of the BMAL1 and CLOCK genes. This relaxation facilitates the binding of RNA polymerase II, thereby enhancing gene transcription. Concurrently, propionic acid reduces the methylation levels in the promoter region of the PER1 gene by modulating the activity of DNA methyltransferase (DNMT3a). This action alleviates epigenetic repression and upregulates PER1 expression. Acetic acid is converted into acetyl-CoA within hepatocytes, serving as a substrate for histone acetylation, which indirectly enhances the transcriptional activity of the BMAL1-CLOCK heterodimer and amplifies the oscillation amplitude of clock genes (65, 66).

Bile acids are crucial metabolites synthesized by the liver that not only participate in lipid digestion and absorption but also directly regulate the expression of clock genes through nuclear receptor-mediated signaling pathways. Primary bile acids, such as cholic acid and ursodeoxycholic acid, specifically bind to the farnesoid X receptor (FXR) located in the hepatocyte nucleus. Upon activation, FXR forms heterodimers with retinoid X receptor (RXR) and directly binds to the FXR response element (FXRE) in the promoter regions of the PER1 and PER2 genes, thereby inhibiting their transcription. *In vivo* experiments demonstrated that injection of the FXR agonist GW4064 in mice led to a decrease of 40 and 35% in the mRNA and protein levels of PER1 and PER2 in liver tissue, respectively. Conversely, FXR knockout mice displayed a significantly enhanced oscillation amplitude of PER gene expression. Secondary bile acids, such as lithocholic acid and deoxycholic acid, bind to the TGR5 receptor on the hepatocyte cell membrane, which activates the cAMP-PKA signaling pathway and promotes the phosphorylation of the transcription factor AP-1. The phosphorylated AP-1 binds to the AP-1 binding site in the promoter region of the BMAL1 gene, thereby enhancing BMAL1 transcription while simultaneously suppressing CRY1 gene expression, thus maintaining the oscillatory balance of the circadian clock (67, 68).

In conclusion, metabolites such as SCFAs and bile acids regulate the circadian rhythms of core clock genes, including BMAL1 and PER, through direct mechanisms such as receptor binding, epigenetic modifications, and activation of signaling pathways.

## 4 Key gut microbes associated with circadian rhythm and MASLD

The interaction between the gut microbiota, circadian rhythms, and hepatic metabolism plays a pivotal role in the progression of MASLD. While numerous microbial taxa are involved in metabolic regulation, certain bacteria exhibit strong rhythmicity and mechanistic links to liver lipid accumulation, inflammation, and fibrosis. This section highlights nine types of gut microbes most relevant to MASLD and circadian biology, with mechanisms of action mediated through short-chain fatty acids (SCFAs), bile acids, gut barrier function, and inflammatory pathways.

### 4.1 Lactobacillus

As one of the dominant short-chain fatty acid (SCFA)–producing bacterial genera in the gut, Lactobacillus primarily produces lactate and acetate, which via cross-feeding are converted to butyrate by lactate-utilizing butyrate producers; these metabolites modulate hepatic metabolism, bile-acid/FXR signalling and intestinal barrier integrity, thereby influencing MASLD development (69, 70).

Under normal circadian conditions, the abundance and metabolic activity of Lactobacillus exhibit daily rhythmicity that is synchronized with feeding patterns (48, 52). For example, in mice, its proliferation and SCFA production peak during the nocturnal active phase, contributing to improved insulin sensitivity, enhanced lipid oxidation, and stimulated secretion of glucagon-like peptide 1 (GLP-1) (71, 72). Moreover, Lactobacillus-derived metabolites can induce the expression of CLOCK and BMAL1 in the liver, thereby maintaining the rhythmic stability of peripheral hepatic clocks (55).

However, under conditions of circadian disruption or high-fat diet, the diurnal oscillations of Lactobacillus are diminished, and its overall abundance declines (73). This leads to impaired bile salt deconjugation, weakened reinforcement of the intestinal mucus barrier, and accelerated progression of metabolic dysfunction-associated steatotic liver disease (MASLD) (74). Experimental studies have demonstrated that supplementation with Lactobacillus strains or specific postbiotics can restore intestinal barrier integrity, reduce lipopolysaccharide (LPS) translocation to the liver, lower systemic inflammation, and attenuate hepatic steatosis (75, 76).

Several *Lactobacillus* strains have improved hepatic steatosis and inflammation in preclinical models. In mice, *L. rhamnosus* GG (LGG) reduced fructose-induced hepatic lipid accumulation and markers of inflammation (e.g., TNF-*α*, IL-1β), while other strains (e.g., *L. delbrueckii* subsp. *bulgaricus*, *L. helveticus*) have been shown to limit diet-induced weight gain and hepatic lipid deposition in experimental models (75, 77). Clinical evidence is supportive but heterogeneous: some observational studies report altered *Lactobacillus* abundance in NAFLD patients, and randomized trials of mixed-strain probiotics (commonly containing *Lactobacillus* spp.) have demonstrated reductions in hepatic fat and serum ALT/AST in select cohorts (78, 79).

Under normal conditions, certain *Lactobacillus* strains contribute substantially to the integrity of the intestinal barrier and host metabolic homeostasis. For example, *Lactobacillus plantarum* has been shown *in vitro* to upregulate expression of tight-junction proteins such as occludin and ZO-1 in human epithelial cell monolayers, increasing transepithelial electrical resistance and suggesting improved paracellular barrier function (80). *In vivo*, administration of *L. plantarum* in human volunteers increased the localization of tight-junction proteins at the epithelial junctions (81). Other studies further demonstrate that *Lactobacillus plantarum* or related strains enhance expression of mucin (e.g., MUC2) and promote mucus layer renewal in murine colon, reinforcing the mucus barrier and limiting luminal antigen or endotoxin translocation (82).

Moreover, certain *Lactobacillus* species produce metabolites with systemic metabolic benefits. A recent study showed that *Lactobacillus acidophilus* supplementation in mouse models of NAFLD–HCC increased portal-vein and hepatic levels of valeric acid (C5:0), and this metabolite reduced hepatic inflammation via inhibition of pathways downstream of Rho-GTPase, implying a role for non-protein microbial metabolites in modulating liver disease progression (83). Meanwhile, *Lactobacillus rhamnosus* GG (LGG) has been demonstrated in high-fructose or high-fat diet–induced steatosis models to mitigate hepatic lipid accumulation, reduce inflammatory cytokines, and improve markers of liver injury, suggesting direct hepatoprotective effects mediated by microbial metabolic activity (83). These results, collectively, support the notion that *Lactobacillus* exerts beneficial effects through both enhancement of gut barrier function and production of bioactive metabolites capable of influencing hepatic glucose and lipid metabolism, inflammation, and overall energy balance.

Therefore, maintaining the rhythmicity and optimal abundance of Lactobacillus is of great significance for MASLD prevention and treatment.

### 4.2 Faecalibacterium

*Faecalibacterium prausnitzii* is a major butyrate-producing member of the healthy human gut microbiota and has emerged as a key contributor to host metabolic homeostasis. Experimental supplementation of *F. prausnitzii* improves hepatic outcomes in diet-induced models: oral administration reduced hepatic triglyceride content, lowered serum transaminases, increased markers of hepatic fatty-acid oxidation and adiponectin signaling, and attenuated adipose tissue inflammation in high-fat or high-fructose rodent models (84). Butyrate itself serves as an important effector: it is oxidized by colonocytes, activates AMP-activated protein kinase (AMPK) in peripheral tissues and the liver to favour *β*-oxidation over lipogenesis, and acts as an epigenetic modulator through histone-deacetylase (HDAC) inhibition — mechanisms that together can strengthen circadian clock gene expression and help re-establish metabolic rhythmicity (55).

Under normal circadian and feeding–fasting cycles, both Faecalibacterium abundance and butyrate production display time-of-day variation that is closely tied to nutrient intake; feeding-timing interventions that restore microbial rhythmicity are associated with improvements in hepatic lipid metabolism in animal models (85). Conversely, circadian disruption, chronic overnutrition or long-term high-fat feeding commonly reduce Faecalibacterium abundance and deplete butyrate, changes that are linked experimentally to impaired gut barrier integrity, shifts in mucosal immune tone, increased translocation of microbial products (e.g., LPS) and worsened hepatic inflammation and fibrogenesis (86). Translationally, *F. prausnitzii* is reported to be lower in patients with NASH/MASLD in several cohorts, and preclinical plus clinical interventions that increase butyrate production — for example dietary fermentable fibers (inulin, fructo-oligosaccharides) or targeted probiotic/next-generation probiotic approaches — can raise Faecalibacterium or butyrate levels and improve markers of hepatic steatosis and systemic inflammation (87, 88).

### 4.3 Akkermansia muciniphila

*Akkermansia muciniphila* is a mucin-degrading commensal that inhabits the intestinal mucus layer and has attracted intense interest as a “next-generation” probiotic because of reproducible links to host metabolic health. Human and animal studies report lower *A. muciniphila* abundance in obesity, metabolic syndrome and in many cohorts of NAFLD/MASLD patients, and reduced levels frequently correlate with worse metabolic and inflammatory parameters (89, 90). Mechanistic work demonstrates several complementary ways by which *A. muciniphila* can protect the gut–liver axis. Surface and secreted proteins (notably Amuc_1100 and the secreted factor P9) interact with host receptors (including TLR2 and ICAM-2) to enhance mucin production, strengthen epithelial tight-junctions and stimulate enteroendocrine GLP-1 release, thereby improving barrier function, incretin signaling and systemic glucose/lipid handling; extracellular vesicles and pasteurized cells retain many of these bioactivities, supporting both live-microbe and postbiotic approaches (91, 92). In multiple rodent models of diet-induced steatosis or NASH, oral administration of selected *A. muciniphila* strains (or pasteurized preparations) reduces body-weight gain, lowers hepatic triglyceride accumulation and inflammation, and in at least one STAM model the human breast-milk isolate AM06—but not a fecal isolate AM02—suppressed progression from NASH to HCC, an effect associated with enhanced hepatic CXCR6^+^ NKT cell responses and reduced macrophage infiltration, illustrating clear strain-specific heterogeneity in therapeutic potential (93).

*A. muciniphila* also influences hepatic metabolism indirectly via its effects on microbially derived metabolites and bile-acid biology. Mucin fermentation yields acetate and propionate (and alters substrate availability for cross-feeding taxa that produce butyrate), actions that can modulate hepatic energy metabolism and bile-acid synthesis as well as stimulate GLP-1/PYY release from L-cells (94); furthermore, several preclinical studies report that *A. muciniphila* or synbiotic combinations reshape bile-acid pools and FXR/TGR5 signaling in ways that favour improved lipid handling (94). Importantly, environmental challenges that perturb host timing and diet—notably obesogenic high-fat feeding and experimental circadian disruption—consistently deplete *A. muciniphila* or impair its functional outputs and thereby exacerbate gut-barrier failure and liver inflammation in animals; however, published time-series data on robust diurnal oscillation of *A. muciniphila* are inconsistent across studies, suggesting that its numerical rhythm may be weak or cohort-dependent while its metabolic activity is nevertheless regulated by feeding–fasting cycles and host circadian state (48, 89). Taken together, the preclinical and early clinical literature supports restoring *A. muciniphila* (or its bioactive components) as a promising strategy to modulate the microbiome–bile-acid–incretin axis and strengthen gut barrier defenses in MASLD, but benefits are strain- and context-dependent and require careful validation in larger, disease-focused clinical trials (95).

### 4.4 Bifidobacterium

*Bifidobacterium* spp. are classical probiotic commensals whose abundance and metabolic output are tightly linked to host feeding behavior and circadian cues. Time-of-day studies show that many gut taxa — including bifidobacteria in several datasets — follow feeding-entrained diurnal oscillations, and interventions that alter host timing (time-restricted feeding) or host melatonin signaling modify bifidobacterial abundance and rhythmicity (96). Functionally, *Bifidobacterium* species produce acetate, propionate and lactate that act as signalling metabolites: these SCFAs engage intestinal receptors such as GPR41/FFAR3 and GPR43/FFAR2 to stimulate incretin release (GLP-1/PYY), modulate energy expenditure and suppress lipogenesis, and — via HDAC inhibition and related epigenetic effects — can influence peripheral clock genes including BMAL1 (97). At the same time, many bifidobacterial strains express metabolic pathways that generate bioactive tryptophan derivatives (for example indole-3-lactic acid) and surface polysaccharides; these molecules activate host receptors (AhR, TLR2) to promote IL-10 production and anti-inflammatory phenotypes in the mucosa, thereby reinforcing barrier function and limiting translocation of pro-inflammatory molecules such as LPS (98).

Loss of Bifidobacterium taxa or their metabolic capacity is a consistent feature of diet- and rhythm-driven models of metabolic disease and is reported in multiple NAFLD/MASLD cohorts, where reductions in bifidobacterial abundance are associated with worse metabolic and inflammatory profiles (99). Preclinical intervention studies demonstrate that selected strains (for example several *B. breve* and *B. longum* isolates) or synbiotic formulations can reduce diet-induced hepatic lipid accumulation, lower serum ALT/AST and systemic inflammatory markers, and restore gut barrier integrity — effects that are mechanistically linked to increased SCFA and ILA production, activation of AMPK/PPARα-dependent hepatic lipid oxidation pathways, and favourable remodelling of bile-acid pools (FXR/TGR5 signalling) (100–102). These data also suggest a translationally attractive concept: circadian-aligned microbial therapies (for example pairing time-restricted feeding with targeted Bifidobacterium supplementation or fermentable-fiber prebiotics) may more effectively restore bifidobacterial rhythmicity and metabolic function and thereby afford greater protection against MASLD than untimed or ad libitum interventions — a hypothesis supported by feeding-timing studies that rescue microbial oscillations and improve hepatic outcomes in rodents.

### 4.5 Bacteroides

Bacteroides is a metabolically versatile and taxonomically diverse genus that sits at a central hub of dietary carbohydrate fermentation, bile-acid biotransformation and host immune crosstalk. Members of this group efficiently metabolize complex polysaccharides released after meals and thereby drive postprandial production of fermentation products such as propionate and acetate, linking feeding–fasting cycles to hepatic substrate availability and signaling (103). Importantly, many *bacteroides* spp. encode bile-salt-hydrolase (BSH) and related enzymes responsible for deconjugation and downstream conversion of primary into secondary bile acids; these microbially produced bile acids act as ligands for FXR and TGR5 and thereby modulate hepatic lipid handling and inflammatory tone (104, 105).

Under physiological conditions Bacteroides abundance and activity are entrained by host feeding rhythms, but this rhythmicity and metabolic output are vulnerable to diet-induced perturbation and circadian disruption. Time-restricted feeding and normal feeding–fasting cycles preserve Bacteroides diurnal dynamics, whereas obesogenic high-fat diets, night-time feeding or experimental circadian misalignment damp these oscillations and remodel bile-acid profiles and SCFA timing (106). When microbial bile-acid transformations are shifted toward excessive formation of hydrophobic secondary bile acids (for example deoxycholic acid), FXR–FGF19 feedback can be impaired and pro-lipogenic and pro-inflammatory hepatic programs are unmasked—an outcome documented in both mechanistic microbiome–bile-acid studies and models of NAFLD (107, 108). Thus, Bacteroides exemplifies a context-dependent regulator of the gut–liver axis: its carbohydrate-fermentative and BSH activities support metabolic homeostasis after normal feeding, but under rhythm disruption or dietary imbalance those same functions can be re-routed into pathways that promote toxic bile-acid accumulation (106), FXR desuppression and hepatic steatosis. Characterizing these bidirectional regulatory modes may enable bile-acid–focused chronotherapies that restore beneficial Bacteroides outputs while avoiding maladaptive conversions (110).

### 4.6 Ruminococcus

Ruminococcus comprises fiber-degrading taxa that are frequently associated with intestinal health and appear to protect against progression of metabolic liver disease in several human cohorts and experimental models. Large cohort metagenomic analyses of biopsy-proven NAFLD found that members of Ruminococcaceae are depleted as fibrosis advances in non-obese patients, and follow-up causal experiments showed that oral administration of a human-derived strain, *Ruminococcus faecis*, reduced serum ALT/AST, diminished collagen deposition and downregulated fibrogenic transcripts (Col1a1, Timp1, *α*-SMA) in multiple mouse NAFLD models (MCD, CDAHFD and db/db), supporting a strain-specific hepatoprotective role (111, 112).

Species within the genus, however, are functionally heterogeneous and must be interpreted at sub-species/strain resolution. *Ruminococcus bromii* acts as a keystone resistant-starch degrader that supplies fermentation substrates to butyrogenic partners (cross-feeding), thereby promoting butyrate production in the community, whereas *Ruminococcus gnavus* specializes in mucin-glycan foraging and has been linked to pro-inflammatory activities in mucosal niches; these divergent ecologies explain why some *Ruminococcus* spp. correlate with protection while others associate with disease. Mechanistically, beneficial Ruminococcus strains are thought to support the liver by increasing SCFA availability (acetate/propionate that feed cross-feeders to produce butyrate), modulating bile-acid composition and FXR-dependent signalling, and strengthening epithelial barrier integrity to limit LPS translocation and systemic inflammation—pathways that together blunt hepatic stellate-cell activation and fibrogenesis. Although daily rhythmicity of Ruminococcus genus members remains underexplored, emerging evidence indicates their abundance and metabolic outputs are responsive to dietary timing and resistant-starch availability, raising the possibility that circadian-aligned dietary or prebiotic interventions could amplify the hepatoprotective functions of favorable *Ruminococcus* strains (113).

### 4.7 Enterobacteriaceae

*Enterobacteriaceae* (for example *Escherichia coli* and *Klebsiella* spp.) are Gram-negative facultative anaerobes that commonly expand when the intestinal ecosystem is stressed by circadian disruption, impaired barrier function or obesogenic diets. Animal models of chronic light-cycle disturbance and jet-lag show shifts in microbial composition with enrichment of Gram-negative, endotoxin-producing taxa and downregulation of barrier-protective species, and multiple clinical and metagenomic studies report increased Proteobacteria/*Enterobacteriaceae* signatures in patients with NAFLD/MASLD. These compositional changes are accompanied by higher portal and systemic concentrations of lipopolysaccharide (LPS) and activation of innate signaling cascades — notably the TLR4–MyD88–NF-κB axis — which drive hepatic production of proinflammatory cytokines (TNF-*α*, IL-6, IL-1β) and contribute to hepatocellular injury in experimental and human disease contexts (58, 114).

Beyond endotoxin signalling, certain *Enterobacteriaceae* (notably high-alcohol-producing *Klebsiella pneumoniae* strains and some *Escherichia* isolates) generate endogenous ethanol in the gut; animal transfer experiments with these isolates reproduce fatty-liver phenotypes and implicate microbially derived ethanol as a pathogenic mediator. Hepatic oxidation of ethanol — principally via alcohol dehydrogenase and the microsomal ethanol-oxidizing system enzyme CYP2E1 — produces acetaldehyde and reactive oxygen species that promote lipid peroxidation, mitochondrial dysfunction and fibrogenic responses, thereby accelerating liver injury. In parallel, LPS and inflammation perturb hepatic clock gene expression and time-of-day responses (for example altering Per and, in specific contexts, BMAL1 expression), which further deranges metabolic rhythmicity and amplifies vulnerability to steatotic progression.

Because Enterobacteriaceae can be causal contributors to MASLD pathogenesis in preclinical systems, several microbial-targeted interventions have been explored. Fecal microbiota transplantation from healthy donors or multi-donor preparations mitigates diet-induced steatosis and hepatic inflammation in mice and has shown encouraging signals in early translational work. Targeted microbiome editing using bacteriophages — notably phages that selectively reduce high-alcohol-producing *Klebsiella pneumoniae* — markedly attenuated liver injury in a recent proof-of-concept study. Separately, engineered probiotic approaches (for example *E. coli* Nissle 1917 strains engineered to deliver hormones or growth factors such as FGF19/IGF-1) have been reported to lower hepatic lipid accumulation in experimental MASLD models. Microbiota transplantation and engineered probiotics collectively demonstrate feasible strategies for inhibiting pathogenic Enterobacteriaceae or replacing their harmful activities. Although most data are derived from preclinical studies, rigorous clinical trials are still needed for validation (109, 115).

### 4.8 Desulfovibrio

*Desulfovibrio* spp. are sulfate-reducing, anaerobic bacteria that produce hydrogen sulfide (H₂S) as a principal metabolic end product (116). Their abundance tends to increase in experimentally induced and diet-driven dysbioses, for example after obesogenic high-fat feeding or in models of circadian disruption, where blooms of sulfur-metabolizing taxa are repeatedly observed. Such expansion is clinically relevant because excess microbially generated H₂S and related sulfur metabolites can erode the mucus layer and perturb tight-junction architecture, thereby increasing intestinal permeability and facilitating translocation of pro-inflammatory microbial products. These mechanistic links are summarized in recent reviews and demonstrated in animal studies showing DSV expansion alongside barrier dysfunction and systemic inflammatory readouts (117).

In experimental models and some patient cohorts, elevated Desulfovibrio correlates with markers of hepatic injury and metabolic stress. Studies report associations between DSV enrichment and higher circulating or portal inflammatory mediators, altered bile-acid profiles, and increased indices of oxidative stress in the liver; mechanistic mouse work further indicates that manipulating DSV levels or sulfur substrate intake modifies liver inflammation and bile-acid homeostasis (118). Importantly, species-level differences exist — not all *Desulfovibrio* isolates behave identically — so causality and net effect can depend on strain identity, diet composition and host context. This nuanced picture is reflected across mechanistic investigations that link SRB activity to hepatic lipid peroxidation, redox imbalance and pro-fibrogenic signalling (117, 119).

Because *Desulfovibrio* expansion is both diet- and rhythm-sensitive, several countermeasures have shown promise in preclinical or small-scale human work. Dietary strategies that reduce sulfur substrate availability (for example reduced sulfur-amino-acid intake) or increase fermentable fiber intake can suppress SRB abundance and H₂S production; specific prebiotics (e.g., glycomacropeptide) and some probiotic regimens have likewise reduced Desulfovibrio and improved barrier and metabolic endpoints in animal studies. Circadian-aware interventions—such as aligning feeding windows to restore host–microbe temporal coupling—also rescue microbial composition and host physiology in models of chronodisruption (116). Together these findings nominate Desulfovibrio as a rhythm-sensitive indicator and modifiable contributor to gut-liver perturbations, while underscoring the need for strain-resolved, mechanistic human studies to validate therapeutic strategies (120).

### 4.9 Alistipes

Alistipes, especially *A. putredinis*, is increasingly recognized as a health-associated member of the human gut community whose depletion is repeatedly observed in cohorts with fatty-liver phenotypes and progressive fibrosis. Clinical metagenomic surveys and case–control studies report lower *Alistipes* abundance in NAFLD/MASLD patients and show negative correlations between *Alistipes* levels and fibrosis severity, suggesting that loss of this genus accompanies metabolic and fibrotic transitions in the liver. Recent experimental work provides functional support for these associations: oral supplementation with an *A. putredinis* strain (Ap77) in a high-fat diet rat model attenuated weight gain, reduced hepatic triglyceride content, lowered serum ALT/AST and pro-inflammatory cytokines, and improved histologic steatosis; these benefits co-occurred with increases in circulating indole derivatives (indole-3-propionic acid, indoleacrylic acid) and butyrate, implying metabolite-mediated host effects (121).

Mechanistically, *Alistipes* exerts much of its influence via tryptophan-derived indole metabolites (notably indole-3-propionic acid, IPA) and, in some strains or via cross-feeding, via short-chain fatty acids such as butyrate (122). IPA and related indoles engage xenobiotic and immune receptors (AhR, PXR), modulate epithelial barrier function and mucosal immune tone, and have been associated in cohort studies with lower fibrosis burden and altered hepatic transcriptomes relevant to stellate-cell activation. Importantly, the IPA–clock interface and circadian biology are increasingly recognized: the microbiota drives diurnal rhythms in tryptophan metabolism and indole production, and AhR-ligand signaling can interact with core clock machinery (AHR ↔ BMAL1/CLOCK), providing a plausible route by which *Alistipes*-derived metabolites influence peripheral circadian programmes that govern hepatic metabolism. That said, the literature also contains context-dependent findings — several experimental studies report that IPA can activate hepatic stellate cells or exacerbate injury in specific fibrogenic models — indicates that the effects of metabolites are associated with differences in dosage, animal models, and disease stages (123). Overall, restoring *Alistipes* abundance or shifting its metabolite output (for example via prebiotics, targeted probiotics or time-of-feeding interventions that re-establish microbial rhythmicity) is a mechanistically plausible strategy to improve gut barrier integrity, re-tune host clock–metabolism coupling, and mitigate MASLD progression, but requires careful validation because of these context-dependent outcomes (7, 123).

## 5 Therapeutic perspectives targeting the circadian–microbiome–liver Axis

Growing evidence supports the notion that targeted interventions along the circadian–microbiome–liver axis have the potential to prevent or reverse metabolic dysfunction–associated steatotic liver disease (MASLD). By realigning behavioral rhythms, restoring microbial balance, and timing medication delivery to match biological cycles, intervention strategies can reinforce host–microbe synchrony and improve metabolic outcomes. This section explores key therapeutic strategies that leverage the reciprocal feedback between circadian biology and gut microbial function to modulate liver health.

### 5.1 Chrononutrition and Behavioral alignment

Chrononutrition focuses on optimizing the timing of food intake to align with endogenous circadian rhythms. Time-restricted feeding (TRF), which limits caloric intake to a consistent daily window during the active phase, has shown substantial promise in restoring gut microbial rhythmicity and metabolic coordination (53, 61). Studies in rodent models demonstrate that TRF enhances the abundance and oscillatory behavior of beneficial taxa such as Lactobacillus and *Faecalibacterium prausnitzii*, increases short-chain fatty acid (SCFA) production, and enhances hepatic expression of clock genes including Bmal1 and Rev-erbα. These changes improve insulin sensitivity, reduce triglyceride accumulation, and decrease inflammatory cytokines (58).

Human trials echo these findings: adopting early time-restricted feeding (eTRF), in which meals are consumed earlier in the day aligned with circadian metabolic peaks, has been associated with reductions in fasting glucose, ALT levels, and hepatic steatosis. Beyond food timing, behavioral strategies such as light exposure regulation, consistent sleep cycles, and physical activity timed to the day promote circadian alignment and support gut microbial and hepatic rhythmicity, underscoring chrononutrition’s role as a foundational MASLD intervention.

### 5.2 Microbiome-targeted probiotics and postbiotics

Probiotic and postbiotic interventions are increasingly considered as precision tools to correct dysbiosis and restore microbial oscillations disrupted in MASLD. Probiotic strains such as Lactobacillus, *Akkermansia muciniphila*, and Bifidobacterium have been shown to improve gut barrier integrity, modulate immune responses, and influence hepatocellular fat metabolism. For example, Akkermansia both degrades and stimulates mucin production, reinforcing epithelial defenses while promoting bile acid signaling through TGR5 activation. Similarly, *Faecalibacterium prausnitzii* produces butyrate, which activates AMPK in hepatocytes, reducing lipid accumulation and inflammation (84, 95).

Postbiotics, such as SCFAs, bacterially derived peptides, or membrane vesicles, bypass the need for bacterial colonization and may be used to directly influence metabolic or immune pathways. Butyrate supplementation, for instance, enhances hepatocyte oxidative metabolism while suppressing lipogenic and pro-inflammatory gene expression, partly via HDAC inhibition and circadian gene activation. Future approaches will likely focus on timing probiotic or postbiotic administration to amplify rhythmic gut–liver metabolic exchanges and improve therapeutic impact (124, 125).

Among the various intervention strategies mentioned in this study, TRF has accumulated some preliminary clinical data support, while probiotic/prebiotic interventions are currently primarily based on preclinical research evidence. Recent small-scale clinical studies have demonstrated that TRF can induce mild weight loss and improve fasting blood glucose and insulin levels in both healthy populations and individuals with metabolic abnormalities (126, 127). The regulatory effects of probiotics and prebiotics on metabolic indicators are predominantly supported by animal studies. In animal models, relevant strains can alleviate obesity and insulin resistance induced by high-fat diets by modulating gut microbiota structure, improving intestinal barrier function, and reducing endotoxemia.

Furthermore, both TRF and probiotic/postbiotic interventions should be comprehensively evaluated based on individual genetic background, lifestyle, underlying diseases, and other factors. Future research should focus on the design of ‘precision interventions’ to enhance the efficacy and safety of intervention strategies.

### 5.3 Dietary modulation for microbial rhythmicity

Diet composition not only determines microbial diversity but also influences the rhythmicity of microbial metabolic activity. Dietary fibers such as inulin and resistant starch drive the expansion of SCFA-producing bacteria (*F. prausnitzii*) and promote their oscillatory SCFA output, critical for lipid metabolism and inflammation control (128, 129). Conversely, high-fat, high-sugar diets disrupt clock-regulated microbial gene expression and favor pathobionts such as Enterobacteriaceae and Desulfovibrio, contributing to gut permeability defects and metabolic endotoxemia (61).

Polyphenol-rich foods and prebiotic supplements have shown positive effects in restoring microbiota-mediated bile acid metabolism and reducing pro-inflammatory taxa. These functional foods interact with microbial pathways and can be additionally potentiated by strategic timing—such as restricting consumption of polyphenol-rich foods to evening periods to support peripheral clock resetting. Incorporating dietary timing with nutritional quality offers an accessible and powerful tool for improving microbiota rhythmicity and liver metabolic resilience (53, 130).

### 5.4 Circadian-timed pharmacotherapy

The emerging field of chronopharmacology posits that the timing of drug delivery modulates efficacy and safety by matching endogenous rhythms in drug targets, transporters, and metabolic pathways. In MASLD, several circadian-sensitive molecular pathways offer logical targets. Farnesoid X receptor (FXR) agonists such as obeticholic acid enhance lipid clearance and bile acid signaling but exhibit time-of-day–dependent effects, with greater efficacy when administered at peak FXR expression (24, 131). Likewise, drugs that activate AMPK or inhibit acetyl-CoA carboxylase (ACC) may be optimized by delivery synchronized to periods of maximal hepatic metabolic turnover (132, 133).

Melatonin has also emerged as an indirect adjunct therapy; by strengthening circadian alignment and reducing oxidative stress, it improves glucose and lipid handling and attenuates hepatocellular injury. The intersection of liver-targeted therapeutics and circadian dynamics opens a new avenue for personalized MASLD treatment based on precise timing of medication administration (134, 135).

### 5.5 Future directions and integrated chrono-microbiome therapies

The “circadian–microbiome–liver axis” provides multiple entry points for innovative drug discovery, clinical trial design, and translational intervention models. Future advances will depend on multi-omics approaches that trace rhythmic interactions across microbial species, host metabolites, and liver transcriptional programs (133). Humanized gut–liver organoid platforms with temporal signaling may enable high-throughput drug screening and predictive modeling. Integrating machine learning and real-time digital health data could guide clinicians in the personalized timing of both nutritional and pharmacological therapies.

The timing-based therapies (such as light modulation and melatonin intervention) and TRF mentioned in this study all aim to regulate circadian rhythms and improve metabolic homeostasis as their core objectives. However, existing research predominantly focuses on population-wide effects, and the impact of individual differences on intervention responses has not been fully elucidated. Factors such as genotype, sleep–wake patterns, and baseline metabolic status can significantly modulate the heterogeneity of intervention effects by altering circadian phase, rhythm amplitude, and metabolic pathway sensitivity. The efficacy of timing-based therapies and TRF is not “universal” but highly dependent on individual characteristics. Future research should incorporate more multidimensional individual stratification metrics and conduct prospective cohort studies to identify optimal intervention protocols for different populations, thereby promoting the transition of circadian rhythm interventions from “population-based” to “individualized” approaches.

By employing multi-omics technologies to capture temporal dynamics and validating intervention effects through organoid models, this study elucidates the molecular mechanisms of circadian microbial interventions. It accurately replicates the molecular processes of circadian interventions *in vitro* while eliminating confounding factors from *in vivo* environments, thereby providing clear target validation for clinical interventions. Future applications may involve real-time monitoring of human circadian signals and microbial dynamics via wearable devices, enabling dynamic adjustment of microbial intervention protocols. This approach could lead to personalized intervention tools, ultimately enhancing clinical compliance and therapeutic efficacy. Ultimately, combining chrononutrition, targeted microbiome modulation, and circadian-timed pharmacotherapy offers a powerful strategy for restoring metabolic homeostasis in MASLD. By addressing not only “what” and “how” but also “when” interventions are applied, future therapeutic paradigms may deliver more effective and sustainable outcomes in metabolic liver disease.

## 6 Conclusion

MASLD represents a multifactorial condition at the intersection of metabolic stress, circadian disruption, and gut microbiome dysregulation. The emerging concept of the circadian–microbiome–liver axis provides a powerful, integrative framework to understand how temporal misalignment at the molecular, cellular, and systemic levels can drive the progression from hepatic steatosis to steatohepatitis and fibrosis.

Throughout this review, we have highlighted how core clock machinery in hepatocytes orchestrates daily rhythms of lipid and glucose metabolism, and how its disruption leads to metabolic inflexibility and heightened vulnerability to dietary insults. Simultaneously, the gut microbiota exhibits its own diurnal rhythmicity, shaped by feeding patterns and host circadian cues. Dysbiosis under circadian misalignment impairs gut barrier function, alters bile acid pools, suppresses short-chain fatty acid (SCFA) production, and facilitates endotoxin translocation—all of which aggravate liver inflammation and lipid accumulation via the gut–liver axis.

Specific bacterial taxa—including *Lactobacillus*, *Faecalibacterium prausnitzii*, *Akkermansia muciniphila*, and *Bifidobacterium*—play rhythm-sensitive roles in modulating gut-liver crosstalk through SCFAs, bile acids, and immunometabolic signaling pathways. Conversely, expansion of opportunistic or inflammatory genera such as Enterobacteriaceae and *Desulfovibrio* under disrupted rhythms drives metabolic endotoxemia and hepatic oxidative stress. Together, these findings position the microbiome as both a mediator and a potential therapeutic target in MASLD.

Therapeutic strategies targeting this axis include chrononutrition and behavioral realignment, microbiome-directed probiotics and postbiotics, functional dietary modulation, and circadian-timed pharmacotherapy. Early human and preclinical studies suggest that aligning food intake, supplement timing, or drug delivery with endogenous biological rhythms enhances both microbiome function and liver metabolic resilience.

Looking ahead, systems biology approaches—coupled with humanized organoid models, real-time circadian monitoring, and machine learning predictions—will enable greater precision in diagnosing, preventing, and treating MASLD. By incorporating the temporal dimension into metabolic liver disease research and clinical care, we can move beyond static views of pathogenesis toward dynamic and personalized interventions that restore synchrony across the circadian–microbiome–liver continuum.
